# First-In-Human safety and long-term exposure data for AAB-003 (PF-05236812) and biomarkers after intravenous infusions of escalating doses in patients with mild to moderate Alzheimer’s disease

**DOI:** 10.1186/s13195-016-0177-y

**Published:** 2016-03-01

**Authors:** Marielle Delnomdedieu, Sridhar Duvvuri, David Jianjun Li, Nazem Atassi, Ming Lu, H. Robert Brashear, Enchi Liu, Seth Ness, James W. Kupiec

**Affiliations:** Pfizer Neuroscience Research Unit, Cambridge, MA USA; Mass General Hospital, Boston, MA USA; Janssen R&D, Spring House, PA USA; Janssen R&D, Fremont, CA USA

**Keywords:** Alzheimer's disease, Pharmacokinetics, Monoclonal antibody, Humans, Safety, Immunotherapy, ARIA

## Abstract

**Background:**

In the First-In-Human (FIH), 39-week, randomized, adaptive design study, safety, tolerability, pharmacokinetics and biomarkers were measured in patients with mild-to-moderate Alzheimer’s disease (AD) after infusion of a humanized monoclonal antibody to amyloid β, AAB-003 (NCT01193608; registered 19 August 2010). AAB-003 was developed by modifying bapineuzumab to reduce Fc-receptor-mediated effector function as a strategy to reduce the removal of amyloid from vessel walls associated with amyloid-related imaging abnormalities with edema/effusions (ARIA-E) without diminishing overall amyloid clearance.

**Methods:**

Eighty-eight patients with AD received up to three infusions of AAB-003 (or placebo) 13 weeks apart at doses of 0.5, 1, 2, 4 or 8 mg/kg in the FIH trial. Dose escalation was based on safety data reviews using a Bayesian escalation algorithm. Subjects who completed the FIH study were permitted to enter a 1-year open-label extension trial with four additional intravenous infusions of AAB-003 (NCT01369225; registered 10 May 2011).

**Results:**

Dose-dependent increases in plasma amyloid β and AAB-003 were observed. No significant changes in cerebral spinal fluid biomarkers were observed. Pharmacokinetics elimination half-life (21–28 days) clearance and volume of distribution values were consistent across dose groups indicating linearity. ARIA-E was the most notable safety finding detected by magnetic resonance imaging (MRI) at 8 mg/kg in two patients. Three cases of microhemorrhage were observed. No new safety findings or MRI abnormalities were observed for the 52 subjects who received AAB-003 in the extension trial.

**Conclusion:**

Based on integrated review of laboratory, electrocardiogram, adverse events, and MRI, AAB-003 was safe and well tolerated up to 8 mg/kg for up to 91 weeks (FIH and extension trials) in patients with mild to moderate AD. Asymptomatic and resolvable ARIA-E was observed after the first or second infusion of AAB-003, similar to bapineuzumab. The AAB-003 dose at which ARIA-E was observed was higher compared to bapineuzumab, supporting the hypothesis that reducing Fc-receptor effector function may reduce the ARIA associated with monoclonal antibodies targeting cerebral amyloid.

**Electronic supplementary material:**

The online version of this article (doi:10.1186/s13195-016-0177-y) contains supplementary material, which is available to authorized users.

## Background

To date, phase 3 immunotherapy trials with humanized monoclonal antibodies (mAb) targeting cerebral amyloid in patients with mild to moderate Alzheimer’s disease (AD) have not shown significant improvements in cognitive or functional outcomes [1, 2]. Treatment-related differences in biomarkers, particularly those in apolipoprotein (APO)ε4 carriers, and reductions in brain amyloid signal by positron emission tomography (PET) imaging indicate mAb-induced central pharmacology [3]. However, these compounds may not have targeted the pertinent amyloid-β (Aβ) species, been given in high enough doses, or may have been administered too late in the progression of AD [2, 4]. The benefits from amyloid immunotherapy may therefore only be realized by introducing these agents much earlier in the course of the disease before significant neurodegeneration is manifest [5].

The incidence of most adverse events in the anti-amyloid mAb trials was comparable between active- and placebo-treated groups and, in general, the compounds were safe and well tolerated [1, 2]. An exception is the observation for some mAbs of abnormalities observed on magnetic resonance imaging (MRI) during safety monitoring [6–9]. The MRI findings, typically identified between the first and third intravenous infusions, were observed on T2-weighted fluid-attenuation inversion recovery (FLAIR) sequences and were initially characterized as vasogenic edema. To more accurately categorize these observations, an expert working group developed the term amyloid-related imaging abnormalities (ARIA) to encompass parenchymal vasogenic edema and sulcal effusions (ARIA-E) and also potential abnormalities observed on GRE/T2* sequences indicative of microhemorrhages and hemosiderosis (ARIA-H) [10].

The incidence of ARIA-E observed in two bapineuzumab phase 3 trials was 15.3 % in the APOε4 carrier study (11.4 % for heterozygotes; 27.3 % for homozygotes at 0.5 mg/kg) and 4.2 % in the noncarrier study (1 mg/kg), respectively. One patient in the placebo group (0.2 %) experienced ARIA-E during each study. On average in these trials, the incidence of asymptomatic ARIA-E among subjects ranged from 45 to 85 % [1]. It is postulated that ARIA is related to an increase in amyloid clearance from cerebral vessels and plaques leading to ARIA-E (vasogenic edema) and microhemorrhage [11]. Although it occurs spontaneously, ARIA is observed more frequently with amyloid-lowering therapy, particularly with antiamyloid immunotherapy [12].

Several lines of evidence support the concept that reduction of Fc-receptor-mediated effector function represents a potential strategy to reduce rapid removal of amyloid from vessel walls without diminishing overall amyloid clearance [13, 14]. In preclinical models, these substitutions reduced antibody-dependent cell-mediated cytotoxicity and complement activation compared to bapineuzumab while still retaining its capacity in amyloid plaque clearance [13]. Such a strategy may improve the safety profile of bapineuzumab (AAB-001), a mAb directed at the N-terminal sequence of Aβ. To achieve this Fc effector function reduction, AAB-003 was developed by introducing a three amino acid mutation in the lower hinge region of bapineuzumab monoclonal antibodies, resulting in the reduced binding of AAB-003 to FcγR compared to bapineuzumab, as well as significantly reduced binding to complement C1q and diminished antibody dependent cellular cytotoxicity (unpublished Janssen data). The AAB-003 pharmacologic profile suggested a reduced risk for ARIA and thereby allowed a higher dose to be assessed compared to bapineuzumab.

The objectives of the First-In-Human (FIH) study were to investigate the safety, tolerability and pharmacokinetics of multiple doses of AAB-003 in subjects with mild to moderate AD. Five doses were studied, with up to three infusions. An important aim was to efficiently establish whether AAB-003 had a better safety profile than bapineuzumab. This study combined single ascending dose (SAD) and multiple ascending dose (MAD) approaches in one adaptive study in which dose escalation and subject allocation were performed based on real-time data capture and prespecified decision rules. The unique design allocated most of the subjects to the highest dose with an acceptable safety profile, while reducing the number of subjects exposed to doses with excessive ARIA-E rates.

Long-term safety and tolerability data were obtained during a 52-week open-label extension (OLE).

## Methods

The FIH study was a randomized, double-blind, placebo-controlled, safety, tolerability, and pharmacokinetic (PK) adaptive design, dose-escalation study of AAB-003 in male and female subjects with mild to moderate AD (NCT01193608). Five AAB-003 dose levels were studied in escalating fashion: 0.5, 1, 2, 4 and 8 mg/kg. In each dose cohort, AAB-003 (or placebo) was administered via a 1-hour intravenous (IV) infusion once every 13 weeks for a total of three infusions (day 1, week 13 and week 26). The study was conducted in accordance with the ethical principles of the Declaration of Helsinki and was in compliance with Good Clinical Practice. A central investigational review board (IRB; Schulman Associates IRB, Cincinnati, Ohio, www.sairb.com) and individual site institutional review boards reviewed and provided approval for the protocols as well as informed consent forms. All subjects provided informed consent and consent for publication.

Subjects who successfully completed the FIH study and continued to meet study inclusion criteria were permitted to enter the 52-week OLE and receive four additional IV infusions of AAB-003 given 13 weeks apart (NCT01369225). Subjects who received active treatment in the FIH remained on the same dose in the OLE study. Subjects who received placebo during the FIH study received AAB-003 in the OLE at a dose based on their dose cohort in the FIH study.

Studies were conducted at 10 centers in the USA and 6 in Korea (see Additional file 1).

Patients between the ages of 50 and 89 years inclusive with a diagnosis of probable AD according to National Institute of Neurological and Communicative Disorders Association criteria were eligible for enrollment. Other requirements included a Mini-Mental State Exam (MMSE) score of 16 to 26 inclusive (12 or greater for OLE), Rosen Modified Hachinski Ischemic score of <4, and an MRI consistent with the diagnosis of AD. Additional study criteria can be found at www.clinicaltrials.gov. Patients with more than one microhemorrhage or lacunar infarct or evidence of a single prior infarct >1 cm^3^, evidence of a cerebral contusion, encephalomalacia, space occupying lesions, or brain tumors were excluded. Patients receiving approved symptomatic treatments for AD were allowed in the FIH study if on stable doses for 4 months prior to screening.

An adaptive dose finding design was employed. The initial cohort size in the FIH study was 4 to 8 subjects (3:1 active:placebo ratio) starting at a 0.5 mg/kg dose of AAB-003. Further increases were approved by an external Data Monitoring Committee (DMC) using a Bayesian escalation algorithm, after review of safety and tolerability data [15]. The algorithm used real-time emerging data on safety endpoints, ARIA-E, and dose-limiting adverse events other than ARIA-E to select the dose for the next cohort. The decision rules to escalate/de-escalate/stay were based on Bayesian posterior probabilities that the proportion of ARIA-E was ≤5 % and the proportion of dose-limiting adverse events other than ARIA-E was ≤30 %. Randomization was to continue until 80 subjects were enrolled (with ~60 allocated to active) or at least 24 subjects were allocated to active treatment at that maximum dose.

The study rules stated that if two or more cases of ARIA-E were observed within a cohort size of 4 to 8, the dose was to be de-escalated and 4 additional subjects allocated to the reduced dose. Further dose allocation was to be reassessed using the Bayesian algorithm and the additional safety experience at the reduced dose (symptomatic case counted as 2). One asymptomatic case of ARIA-E would have led to maintaining the current dose and allocating another 4 subjects to it; further dose allocation was to be reassessed based on observed safety. To generate additional FIH safety and biomarker data, 12 more subjects (10 active, 2 placebo) were enrolled at the 2 or 4 mg/kg doses once either dose was deemed safe per Bayesian escalation algorithm. Patients who completed the FIH without drug-related serious adverse events (SAE) and met inclusion criteria were permitted to enter a 52-week OLE study to provide longer term safety and tolerability data.

### Safety

Safety assessments, including adverse event (AE) reports, physical and neurological examinations, suicidality assessments, 12-lead electrocardiograms (ECGs), and laboratory determinations, were performed at screening, baseline and at predetermined visits for both FIH and OLE studies. Vital signs were obtained at all visits. During and after infusion of study drug, patients were observed for 6 hours. Sitting blood pressure and pulse were measured at 15-min intervals during the infusion, for the first hour after infusion and every 30 min thereafter. Based on the bapineuzumab clinical trial experience, FIH patients were monitored for MRI brain abnormalities (ARIA-E, ARIA-H) at screening (baseline scan), 3 and 6 weeks after the first and second infusions, and 6 weeks following the third and last infusion [6, 16]. No MRI volumetric analysis was performed, nor was amyloid PET imaging performed. In the OLE study, MRIs were obtained 6 weeks after each infusion and at week 52. The study treatment duration was 39 weeks for the FIH and 52 weeks for the OLE and included a final safety follow-up visit. All subjects who received an infusion of study medication (including partial infusions) were included in the safety analysis population.

### PK and PD in the FIH Study

#### Serum AAB-003, Plasma Aβ_x-40_ and anti-AAB-003 antibodies

Standard, model-independent, noncompartmental PK methods were used to characterize the serum concentration–time profiles of AAB-003 and plasma Aβ_x-40_. Standard PK parameters were derived from the concentration–time data following AAB-003 administration: peak concentration (C_max)_, time to C_max_ (T_max)_, total area under the concentration–time curve to 13 weeks (AUC_0-13weeks_) and infinity (AUC_inf_), apparent terminal phase elimination half-life (t_1/2_), systemic clearance (CL) and steady-state volume of distribution (Vss). Blood samples for serum AAB-003 PK evaluations in the FIH study were collected on day 1 (predose, 1 hour (end of infusion), 1.5, 2, 4 and 6 hours postinfusion start), day 2 (24 hours postinfusion start), weeks 1, 3, 6, 10, 13 (predose, 1 hour (end of infusion)), week 19, week 26 (predose, 1 hour (end of infusion), 1.5, 2, 4, 6, and 24 hours postinfusion start), and weeks 32 and 39.

Blood samples for exploratory analysis of plasma Aβ_x-40_ were collected from all patients on day 1 (predose, 1 hour (end of infusion) and 2 hours postinfusion start), day 2, weeks 1, 6, and 13 (predose, 1 hour (end of infusion) and 2 hours postinfusion start), week 19, week 26 (predose, 1 hour (end of infusion) and 2 hours postinfusion start), week 32 and week 39. Intensive blood sampling was implemented after the first and third doses in the FIH study to evaluate AAB-003 PK and plasma Aβ_x-40_ after a single dose and at steady state. In the OLE study, PK and pharmacodynamics (PD) samples were collected less frequently. Therefore, PK and PD data presentation and discussion is limited to the FIH study. Serum samples for anti-AAB-003 antibodies were collected prior to dosing in the FIH study at weeks 13, 26 and 39.

The PK/PD analysis population included all randomized subjects who received at least one infusion of study medication and had at least one postdose PK/PD assessment.

### Cerebrospinal Fluid Biomarkers in the FIH study

Cerebrospinal fluid (CSF) samples were collected at baseline and at approximately week 32 (or early withdrawal) from a subset of subjects who provided consent from the 2 mg/kg, 4 mg/kg and 8 mg/kg cohorts in the FIH. CSF samples were analyzed for Aβ_x-40_, Aβ_x-42_, t-tau, p-tau and AAB-003 concentrations. CSF sample collection was only made mandatory in the FIH after reaching the highest dose of 8 mg/kg. No more than 5 patients provided consent for CSF collection at week 45 of the OLE. Due to this small number of CSF samples, CSF exposure and biomarker data are limited to the FIH.

The CSF analysis population consisted of subjects in the 8 mg/kg AAB-003 cohort who provided sufficient CSF samples at baseline and 32 weeks to allow for the assaying of both samples, and who had no ARIA-E occurrence. There were insufficient numbers of subjects on AAB-003 in the 2 mg/kg and 4 mg/kg cohorts for group analysis. Placebo data from the 2 mg/kg and 4 mg/kg cohorts were included in the analysis. The change from baseline for each of the CSF biomarkers in the 8 mg/kg AAB-003 cohort was analyzed using one-sample paired *t*-test. To compare AAB-003 and placebo-treated subjects, an exploratory analysis using a mixed model with fixed effects for treatment, center, age, baseline MMSE category, baseline biomarker value and random subject effect was also performed.

### Assay Methods

All assays were conducted using validated methodologies developed in compliance with Janssen AI Bioanalytical Development operating procedures and documented in method validation reports. Enzyme-linked immunosorbent assay methodologies were used to measure AAB-003 and anti-AAB-003 antibody concentrations in serum and t-tau and p-tau concentrations in CSF (Innotest, Fujirebio). CSF AAB-003 concentrations were determined using an electrochemiluminescence methodology. Plasma Aβ_x-40_ samples, CSF Aβ_x-40_ and Aβ_x-42_ concentrations were determined by Meso Scale Discovery technology and plate reader.

### Efficacy Endpoints in the FIH and OLE studies

Exploratory efficacy of AAB-003 was evaluated using the Alzheimer's Disease Assessment Scale-Cognitive Subscale (ADAS-Cog), Disability Assessment for Dementia (DAD), Neuropsychiatric Inventory (NPI), Clinical Dementia Rating (CDR) and MMSE. These endpoints were summarized by treatment group based on a full analysis population set consisting of all randomized patients who received at least one infusion of study medication.

## Results

### Patients

**Fig. 1:**
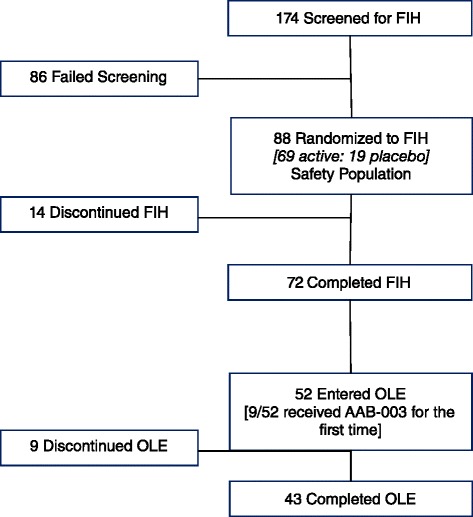
Patient disposition. *FIH* First-In-Human, *OLE* Open label extension

A total of 174 patients were screened for participation in the FIH study of which 88 were confirmed as eligible, randomized to treatment and constituted the safety population (Fig. 1). Patient ages ranged from 51 to 88 years and the mean ages across treatment groups were comparable and ranged from 64.5 to 71.5 years (Table 1). Subjects were predominantly White (47 %) or Asian (43 %) and to a lesser extent Black (10 %). None of the participating patients were known to have any illness at baseline that might interfere with the activity of AAB-003 or interpretation of the study results. Many enrolled patients had a history of chronic, stable medical conditions that did not interfere with the conduct of this study. All patients, except for 4, took at least one concomitant medication. The most commonly reported concomitant medications were donepezil (39 %), memantine (32 %) and acetylsalicylic acid (35 %). Of the 72 subjects who completed the FIH study, 52 enrolled in the OLE (19 males and 33 females). Patients in the OLE were predominantly White or Asian and mostly ranged in age from 52 to 74 years (Table 2).

**Table 1:** Demographic and baseline characteristics—First-In-Human

Number of subjects	AAB-003					
	0.5 mg/kg (N = 6)	1 mg/kg (N = 6)	2 mg/kg (N = 16)	4 mg/kg (N = 17)	8 mg/kg (N = 24)	Placebo (N = 19)
Age (years), mean (SD)	64.5 (6.4)	67.2 (7.4)	70.7 (10.8)	71.5 (8.8)	64.5 (7.6)	71.1 (7.6)
Sex, n						
Male	2	3	2	8	10	12
Female	4	3	14	9	14	7
Ethnicity, n						
White	4	3	5	6	12	11
Black	2	2	1	0	1	3
Asian	0	1	10	11	11	5
Weight (kg) mean (SD)	70.9 (13.4)	69.8 (18.4)	58.0 (10.7)	64.1 (10.5)	70.8 (17.5)	74.1 (21.8)
MMSE score, mean (SD)	22.7 (1.37)	22.5 (2.43)	20.8 (3.71)	20.1 (3.00)	21.2 (2.82)	21.2 (3.78)

*MMSE* Mini-Mental State Exam, *SD* Standard deviation

**Table 2:** Demographic and baseline characteristics—52-week Open Label Extension

Number of subjects	AAB-003					
	0.5 mg/kg (N = 6)	1 mg/kg (N = 3)	2 mg/kg (N = 12)	4 mg/kg (N = 10)	8 mg/kg (N = 12)	Placebo to AAB-003 (N = 9)*
Age (years), mean (SD)	65.2 (6.5)	63.0 (7.8)	68.8 (10.6)	71.3 (8.8)	60.7 (5.8)	71.7 (7.9)
Sex, n						
Male	2	1	2	5	4	5
Female	4	2	10	5	8	4
Ethnicity n						
White	4	1	2	1	3	4
Black	2	2	0	0	2	0
Asian	0	0	10	9	7	5
Weight (kg), mean (SD)	71.8 (13.7)	80.0 (26.2)	54.0 (8)	65.0 (12.8)	69.9 (18.0)	66.7 (22.3)
MMSE, mean (SD)	24.7 (1.86)	21.0 (4.36)	19.3 (5.05)	18.5 (4.86)	21.3 (5.40)	18.9 (5.64)

*Number of placebo subjects who transitioned to AAB-003 in the open label extension: 1 at 0.5 mg/kg, 1 at 1 mg/kg, 3 at 2 mg/kg, 1 at 4 mg/kg, and 3 at 8 mg/kg. *MMSE* Mini-Mental State Exam, *SD* Standard deviation

### Safety

Of the 69 patients who received AAB-003 in the FIH study, 42 (61 %) reported a treatment-emergent adverse event (TEAE) (Table 3). Of the 19 patients treated with placebo, 12 (63 %) reported a TEAE. There were 14 discontinuations from the FIH study: 9 no longer willing to participate, 3 lost to follow-up, 1 travel inconvenience, and 1 cerebral microhemorrhage. The most commonly reported all-causality TEAEs for combined AAB-003-treated subjects were decreased appetite (7 patients, 10.1 %) followed by dizziness and headache (5 patients each, 7.2 %). Two patients who received placebo reported dizziness (10.5 %) and 1 reported headache (5.3 %). The most commonly reported treatment-related TEAE was ARIA-H (3 patients, 4.3 %), ARIA-E (n = 2, 2.9 %) and nausea (n = 2, 2.9 %). The majority of TEAEs were mild to moderate in severity; no deaths were reported. At the highest dose, the frequency and severity of TEAEs tended to increase. The more severe treatment-related AEs (6 moderate, 1 severe and 1 very severe) at 8 mg/kg were predominantly related to the central nervous system: ARIA-E (n = 2, 8.3 %), ARIA-H (n = 2; 8.3 %), headache (n = 1, 4.2 %), insomnia (n = 1, 4.2 %), dizziness (n = 1, 4.2 %), confusional state (n = 1, 4.2 %) and impaired self-care (n = 1, 4.2 %). Of the 9 SAEs reported for 9 patients in the FIH, 5 were treatment related: 2 ARIA-E and 3 ARIA-H (one ARIA-H reported as an SAE was not reported as an AE). None of the unrelated SAEs were concurrent with ARIA findings.

**Table 3:** Treatment-emergent adverse events occurring in ≥2 patients in any treatment group––First In Human

Adverse event	AAB-003					
	0.5 mg/kg (N = 6)	1 mg/kg (N = 6)	2 mg/kg (N = 16)	4 mg/kg (N = 17)	8 mg/kg (N = 24)	Placebo (N = 19)
	n (%)	n (%)	n (%)	n (%)	n (%)	n (%)
Anxiety	0	0	2 (12.5)	0	0	2 (10.5)
ARIA-E	0	0	0	0	2 (8.3)	0
ARIA-H	0	0	1 (6.3)	0	2 (8.3)*	0
Back pain	0	0	2 (12.5)	0	1 (4.2)	0
Decreased appetite	0	2 (33.3)	3 (18.8)	2 (11.8)	0	0
Dizziness	0	0	2 (12.5)	2 (11.8)	1 (4.2)	2 (10.5)
Fall	0	0	2 (12.5)	1 (5.9)	0	1 (5.3)
Gait disturbance	0	0	1 (6.3)	2 (11.8)	0	1 (5.3)
Headache	1 (16.7)	1 (16.7)	0	2 (11.8)	1 (4.2)	1 (5.3)
Nasopharyngitis	1 (16.7)	0	1 (6.3)	0	2 (8.3)	1 (5.3)
Nausea	1 (16.7)	0	1 (6.3)	0	2 (8.3)	1 (5.3)
Sinusitis	0	1 (16.7)	0	0	2 (8.3)	0
Urinary tract infection	1 (16.7)	0	1 (6.3)	0	2 (8.3)	0
Weight increased	0	0	0	0	2 (8.3)	0
Vomiting	0	0	0	1 (5.9)	2 (8.3)	1 (5.3)

*One case was not reported as an adverse event (ARIA-H incidence based on central magnetic resonance imaging reporting was 12.5 %). *ARIA-E* Amyloid-related imaging abnormalities with edema/effusions, *ARIA-H* Amyloid-related imaging abnormalities indicative of microhemorrhages and hemosiderosis

Asymptomatic ARIA-E was identified in 2 (8.3 %) of the 24 patients who received 8 mg/kg. The first case was detected at the week 6 scheduled MRI scan and the study drug was immediately discontinued, as per protocol. An increased area of ARIA-E was noted in a subsequent MRI scan 22 days later. Thereafter, ARIA-E showed progressive resolution on MRI at weeks 16 and 19, with complete resolution reported on MRI at week 39. The subject was ApoE4 negative (ε3/ε3). The second case was identified on MRI at week 16 (3 weeks after second infusion) and the study drug was discontinued. Thereafter, an increased area of ARIA-E was observed on MRI at week 19. Complete resolution was reported on MRI at week 32. The subject was ApoE4 positive (ε3/ε4). There was no clear link between ARIA-E and ApoE4 carrier status in this study.

A total of 4 asymptomatic ARIA-H cases were observed: 1 (6.3 %) in the 2 mg/kg treatment group and 3 (12.5 %) in the 8 mg/kg treatment group. The patient in the 2 mg/kg treatment group entered the trial with a single ARIA-H which increased to three lesions post-treatment initiation. One case at 8 mg/kg was concomitant with the ARIA-E at week 6 described above; it remained unchanged on subsequent MRIs. The second 8 mg/kg case involved a single ARIA-H detected on the scheduled MRI at week 16 following two doses of study drug. An additional ARIA-H was reported on MRI at week 32. The study drug was not stopped and the event outcome was not considered resolved at the end of the study. The third ARIA-H was observed at the week 32 visit and reported as an SAE (but not as an AE).

Of the 52 patients who received AAB-003 in the OLE study, 40 (75 %) reported a TEAE (Table 4), the majority of which were mild to moderate in severity. No deaths were reported. There were 9 discontinuations (17 %), none considered related to the study drug. The most commonly reported all-causality TEAEs were depression (n = 6, 11 %) followed by urinary tract infection (n = 5, 9 %). Treatment-related TEAEs were reported by 12 patients (23 %), 6 of whom were in the 8 mg/kg treatment group. All other treatment groups had similar number of treatment-related TEAEs. No findings of ARIA-E or ARIA-H were identified in the OLE. Of the 11 SAEs reported for 8 patients in the OLE, none were treatment related.

**Table 4:** Treatment-emergent adverse events occurring in ≥2 patients in any treatment group—52-week open label extension

Adverse event	AAB-003					
	0.5 mg/kg to 0.5 mg/kg (N = 6)	1 mg/kg to 1 mg/kg (N = 3)	2 mg/kg to 2 mg/kg (N = 12)	4 mg/kg to 4 mg/kg (N = 10)	8 mg/kg to 8 mg/kg (N = 12)	Placebo to AAB-003* (N = 9)
	n (%)	n (%)	n (%)	n (%)	n (%)	n (%)
Anxiety	0	0	2 (16.7)	0	1 (8.3)	0
Back pain	0	0	2 (16.7)	0	0	1 (11.1)
Depression	0	0	1 (8.3)	0	3 (25)	2 (22.2)
Dizziness	0	0	1 (8.3)	0	2 (16.7)	1 (11.1)
Contusion	0	0	0	0	2 (16.7)	0
Fall	1 (16.7)	2 (66.7)	0	0	1 (8.3)	0
Pollakiuria	0	0	2 (16.7)	0	0	0

*Number of placebo subjects who transitioned to AAB-003 in the open label extension: 1 at 0.5 mg/kg, 1 at 1 mg/kg, 3 at 2 mg/kg, 1 at 4 mg/kg, and 3 at 8 mg/kg

For both the FIH and OLE, there was no evidence of clinically significant changes in laboratory tests, vital sign measurements, physical examination, neurologic examination, or ECG results.

### PK and PD for the FIH

**Fig. 2:**
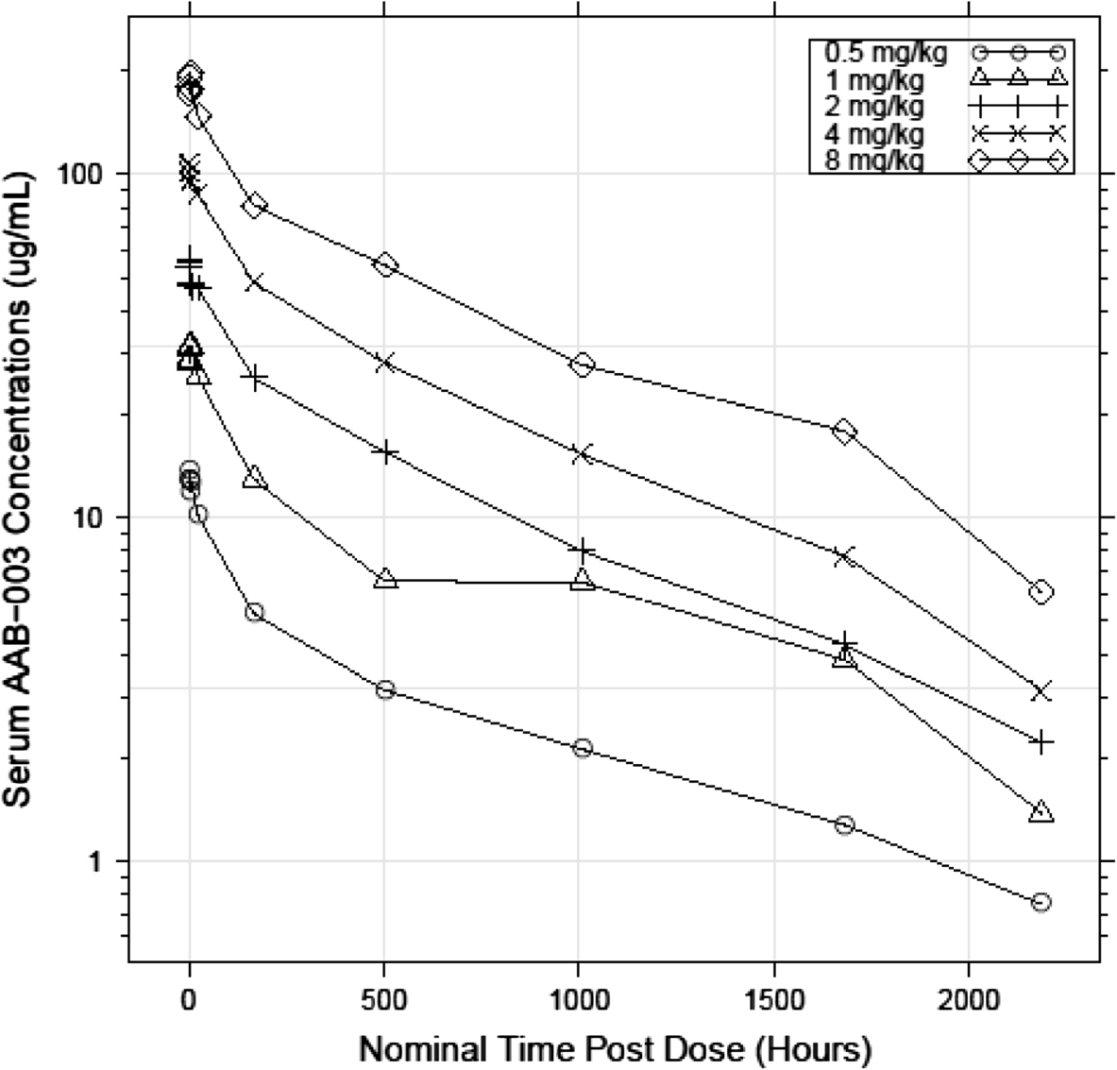
Mean serum AAB-003 concentrations following initial intravenous infusions of increasing doses of AAB-003

Mean AAB-003 serum concentration versus time curves for each dose are presented in Fig. 2. AAB-003 serum C_max_ and AUC_inf_ values after single doses increased with dose in a roughly dose-proportional fashion within the 0.5–8 mg/kg dose range. The t_1/2_ was similar across all dose groups and was approximately 21–28 days. The median T_max_ values after the infusion ranged from 1.5–4.0 hours across doses. AAB-003 clearance (CL) and volume of distribution (Vss) values were consistent across all dose groups suggesting linear PK (Table 5). The mean (%CV) CSF AAB-003 concentration following the 8 mg/kg dose was 79.16 (67) ng/ml.

**Table 5:** Summary of serum AAB-003 pharmacokinetic parameters

Parameters (units)	0.5 mg/kg (N = 6)	1 mg/kg (N = 6)	2 mg/kg (N = 16)	4 mg/kg (N = 17)	8 mg/kg (N = 24)
C_max_ (μg/ml)	13.79 (48)	32.05 (33)	58.74 (21)	122.4 (14)	223.7 (28)
T_max_ (hours)	1.92 (1.50, 6.05)	4.00 (2.17, 6.02)	1.54 (1.20, 2.12)	1.50 (1.00, 24.0)	3.02 (1.07, 6.00)
AUC_inf_ (μg/hour/ml)	5597 (39)	13060 (18)	25270 (17)	46430 (21)	82140 (23)
t_1/2_ (days)	27.7 ± 6.99	20.04 ± 8.92	24.45 ± 4.87	21.10 ± 5.20	22.89 ± 4.51
CL (ml/hour/kg)	0.0893 (39)	0.0766 (18)	0.0791 (17)	0.0862 (21)	0.0974 (23)
Vss (ml/kg)	78.44 (38)	59.74 (37)	61.67 (21)	60.44 (26)	75.12 (27)

N: Number of patients with pharmacokinetic parameters on day1 (may be different from number of patients with estimable t_1/2_). Geometric mean (geometric %CV) for all except median (range) for T_max_ and arithmetic mean ± standard deviation for t_1/2_. *AUC*_*inf*_ Total area under the concentration–time curve to infinity, *CL* Systemic clearance, *C*_*max*_ Observed peak concentration, *t*_*1/2*_ Apparent terminal phase elimination half-life, *T*_*max*_ Time to peak concentration, *Vss* Steady-state volume of distribution

Following AAB-003 infusion, a dose-dependent increase in plasma Aβ_x-40_ was observed across the entire dose range. The median time to maximum plasma Aβ_x-40_ increased from approximately 24 hours at lower doses (0.5–2 mg/kg) to approximately 164 hours at higher doses (4–8 mg/kg) (Table 6). The t_1/2_ for plasma Aβ_x-40_, when estimated, was approximately 30 days. No anti-AAB-003 antibodies were observed in the FIH study.

**Table 6:** Summary of plasma Aβ_x-40_ pharmacokinetic parameters

Parameters (units)	Placebo (N = 19)	0.5 mg/kg (N = 6)	1 mg/kg (N = 5)	2 mg/kg (N = 14)	4 mg/kg (N = 16)	8 mg/kg (N = 22)
C_max_ (ng/ml)	0.3511 (25)	3.321 (50)	6.959 (29)	8.202 (19)	11.89 (10)	18.03 (19)
T_max_ (hours)	168 (0.00, 2540)	24.2 (22.3, 149)	24.0 (24.0, 169)	24.0 (24.0, 170)	166 (24.0, 238)	164 (92.2, 2050)
AUC_0-13weeks_ (ng/hour/ml)	631.6 (23.3)	2647 (31)	6057 (47)	7854 (19)	12,940 (17)	22,820 (25)
t_1/2_ (days)	Not Reported	26.57*	32.14*	30.71 ± 3.23	25.4, 33.5*	Not reported

N: Number of patients with pharmacokinetic parameters on Day1 (may be different from number of patients with estimable t_1/2_. Geometric mean (geometric %CV) for all except median (range) for T_max_ and arithmetic mean ± standard deviation for t_1/2_. *Individual values for n < 3. *AUC*_*0-13weeks*_ Total area under the concentration–time curve to 13 weeks, *C*_*max*_ Observed peak concentration, *t*_*1/2*_ Apparent terminal phase elimination half-life, *T*_*max*_ Time to peak concentration

### CSF biomarkers (CSF Aβ_x-40_, Aβ_x-42_, tau, p-tau)

Results of a one-sample paired *t*-test used to compare the change in CSF biomarkers of patients in the 8 mg/kg treatment group at baseline and at week 32 are detailed in Table 7. The change from baseline in the 8 mg/kg treatment group was (in pg/ml): 113.53 for Aβ_x-40_ (placebo = 348.64, n = 5), 48.85 for Aβ_x-42_ (placebo = 24.04), –15.26 for tau (placebo = 67.94), and 1.23 for p-tau (placebo = 1.92). The within-group change from baseline to week 32 for all CSF biomarkers—Aβ_x-40_, Aβ_x-42_, tau and p-tau—in the 8 mg/kg treatment group did not demonstrate statistical significance.

**Table 7:** One-sample paired *t*-test for change from baseline to week 32 of cerebrospinal fluid biomarkers in the 8 mg/kg group

Biomarker	Mean change	80 % CI	95 % CI	*P* value
Aβ_x-40_ (pg/ml)	113.53	(–170.30, 397.35)	(–339.06, 566.12)	0.599
Aβ_x-42_ (pg/ml)	48.85	(–11.79, 109.48)	(–47.84, 145.53)	0.297
Tau (pg/ml)	−15.26	(–53.54, 23.02)	(–76.30, 45.78)	0.600
P-tau (pg/ml)	1.23	(–1.89, 4.35)	(–3.74, 6.21)	0.603

CI Confidence interval

The mixed model analysis showed the difference in change from baseline to week 32 between the 8 mg/kg treatment and placebo groups was not statistically significant (at level 0.05) for any CSF biomarkers.

### Exploratory efficacy endpoints for the FIH and extension study

Neither study was designed to compare between-treatment groups or to assess the overall efficacy of AAB-003. Efficacy evaluations were thus limited to calculation of the mean change from baseline of each exploratory efficacy endpoint calculated at weeks 13, 26 and 39 for the FIH and at weeks 13, 26, 39 and 52 for the OLE. Based on inspection of the mean changes from baseline in ADAS-Cog, DAD, NPI, CDR and MMSE among the treatment groups, there was no clear indication of drug effect over time in either study. This is anticipated as the number of subjects is small in both studies.

## Discussion

During the 39-week FIH study, the only notable safety findings were observed by MRI at 8 mg/kg and included two cases of asymptomatic ARIA-E observed at week 6 and week 16 (both resolved by week 32), and three new microhemorrhages (confirmed by being observed on more than one MRI scan). Based upon all laboratory results, ECG data, integrated assessments of AE, SAE and MRI findings, AAB-003 was found to be safe and well tolerated up to 8 mg/kg in the FIH study. In the 52-week OLE no new safety or tolerability findings emerged and no MRI findings of ARIA-E or ARIA-H were reported at any dose. The absence of ARIA-E in the OLE, particularly for the 8 mg/kg dose, is consistent with the bapineuzumab experience showing ARIA-E most likely to appear after the first or second infusion of drug.

The FIH study was also designed to assess the concept that modifying bapineuzumab to reduce Fc-receptor-mediated effector function would reduce the risk for ARIA-E and microhemorrhage. The results from this FIH study appear to support that concept: the dose associated with ARIA-E in this trial was 8 and 16 times higher compared to bapineuzumab in the phase 3 APOε4 carrier and noncarrier studies, respectively [1].

The pharmacokinetics of AAB-003 following IV infusion every 13 weeks were characterized by slow clearance and low volume of distribution which is typical of monoclonal antibodies including bapineuzumab [6]. AAB-003 C_max_ and AUC_inf_ values increased in a dose-proportional fashion with relatively similar CL and Vss values across the dose groups (0.5–8 mg/kg), suggesting linear pharmacokinetics. Accumulation of AAB-003 concentrations was minimal with every 13-week infusion (data not shown). Average CSF AAB-003 concentrations at week 32 were less than 1 % of the observed serum AAB-003 concentrations. This is also typical of other monoclonal antibodies [17]. Similar to bapineuzumab, no anti-AAB-003 antibodies were observed indicating low AAB-003 immunogenicity.

A dose-dependent increase in plasma Aβ_x-40_ concentrations demonstrated peripheral target engagement. Plasma Aβ_x-40_ levels were similar to those observed after bapineuzumab administration at comparable doses (Pfizer Unpublished Data on File). The lack of significant change in CSF levels of Aβ_x-40_ and Aβ_x-42_, tau and p-tau may be due to the short duration of therapy (32 weeks) or the smaller sample size. Further investigation beyond 32 weeks is necessary to accurately delineate the effect of AAB-003 on CSF biomarker of AD.

By combining sequential SAD and MAD studies into one adaptive study design, the overall number of patients and time required to characterize the dose-limiting adverse event of special interest (ARIA-E) was reduced. The Bayesian algorithm and predefined decision rules efficiently allowed advancement from the lowest dose of 0.5 mg/kg up to the highest dose of 8 mg/kg in smaller cohort sizes, while allocating the largest number of patients to the highest dose of 8 mg/kg where ARIA-E emerged. The use of real-time safety data collection allowed these decisions to be made by the DMC with only brief pauses to the trial during evaluation. Although not utilized during the conduct of the study, the predecision rules on dose reduction and patient allocation provided clear guidance to the DMC and study team on how to continue the study and generate additional information in the event an unacceptable number of dose-limiting AEs had been observed.

The limited 39-week duration for the FIH was based on clinical experience with bapineuzumab indicating ARIA-E was most likely to occur after the first or second infusions given 13 weeks apart. Consequently, there are two limitations to the data provided from the combined experience from the FIH and 1-year extension trial. First, the confidence in the true rate of ARIA-E observed at 8 mg/kg, an association with APOε4 status, and the risk for coincident ARIA-H, are limited by the relatively small sample size of 24 patients at that dose in the FIH study. Second, the safety profile of AAB-003 in patients who experienced ARIA-E and continued to receive AAB-003 is unknown, since dosing was discontinued, per protocol, for the two patients with ARIA-E in the FIH study. Future AAB-003 clinical studies of larger size, along with a consideration to permit continued dosing of AAB-003 in patients who experience ARIA-E, would address these limitations.

## Conclusion

The results from the FIH and extension study showed AAB-003 was safe and well tolerated up to 8 mg/kg for up to 91 weeks (First-In-Human study and its open label extension) in patients with mild to moderate AD. Asymptomatic and resolvable ARIA-E was observed after the first or second infusion of AAB-003, which is similar to bapineuzumab. In contrast, the AAB-003 dose at which ARIA-E was observed was considerably higher compared to bapineuzumab, a finding that supports the hypothesis that reducing Fc-receptor effector function may reduce the risk of ARIA associated with monoclonal antibodies targeting cerebral amyloid.

## Additional file

Additional file 1:
**List of investigators and corresponding ethics committees or institutional review boards.** (DOC 60 kb)

## Abbreviations

AAB-001: Bapineuzumab
AD: Alzheimer’s disease
ADAS-cog: Alzheimer's Disease Assessment Scale-Cognitive Subscale
AE: Adverse event
Apo: Apolipoprotein
ARIA-E: Amyloid-related imaging abnormalities with edema/effusions
ARIA-H: Amyloid-related imaging abnormalities indicative of microhemorrhages and hemosiderosis
AUC_0-13weeks_: Total area under the concentration–time curve to 13 weeks
AUC_inf_: Total area under the concentration–time curve to infinity
Aβ: Amyloid β
CDR: Clinical dementia rating
CL: Systemic clearance
C_max_: Observed peak concentration
CSF: Cerebrospinal fluid
DAD: Disability Assessment for Dementia
DMC: Data Monitoring Committee
ECG: Electrocardiogram
FIH: First-In-Human
FLAIR: Fluid-attenuation inversion recovery
IRB: Investigational review board
IV: Intravenous
mAb: Humanized monoclonal antibodies
MAD: Multiple ascending dose
MMSE: Mini-Mental State Exam
MRI: Magnetic resonance imaging
NPI: Neuropsychiatric inventory
OLE: Open label extension
PD: Pharmacodynamic
PET: Positron emission tomography
PK: Pharmacokinetic
p-tau: Phosho-tau
SAD: Single ascending dose
SAE: Serious adverse events
t_1/2_: Apparent terminal phase elimination half-life
TEAE: Treatment-emergent adverse event
T_max_: Time to peak concentration
Vss: Steady-state volume of distribution
