# Supplementary material for: First-In-Human safety and long-term exposure data for AAB-003 (PF-05236812) and biomarkers after intravenous infusions of escalating doses in patients with mild to moderate Alzheimer’s disease
Source: Alzheimers Res Ther. 2016 Mar 1;8:12. doi: 10.1186/s13195-016-0177-y (PMC4772335; doi:10.1186/s13195-016-0177-y)
Supplement: Additional file 1: — List of investigators and corresponding ethics committees or institutional review boards. (DOC 60 kb) [file 13195_2016_177_MOESM1_ESM.doc]

# LIST OF INVESTIGATORS AND CORRESPONDING ETHICS COMMITTEES OR INSTITUTIONAL REVIEW BOARDS

## Republic Of Korea

| **Principal Investigator** | **Address(es)** | **Institutional Review Board or Ethics Committee Address(es)** |
| --- | --- | --- |
|  |  |  |
| Jae-Hong Lee | ASAN Medical Center  86, Asanbyeongwon-gil, Songpa-gu  Seoul, 138-736  KOREA, REPUBLIC OF | Asan Medical Center IRB  88, Olympic-ro, 43-gil, Songpa-gu  Seoul, 138-736  KOREA, REPUBLIC OF |
|  |  |  |
| Dr. Duk L. Na | Samsung Medical Center, Department of Neurology  50 Irwon-Dong, Gangnam-Gu  Seoul, 135-710  KOREA, REPUBLIC OF | HRPP(Human research protection Program) Help Desk  50, Irwon-dong, Gangnam-Gu  Seoul, 135-710  KOREA, REPUBLIC OF  Samsung Medical Center Institutional Review Board  Samsung Medical Center Institutional Review Board  50 Irwon-Dong, Gangnam-Gu  Seoul, 135-710  KOREA, REPUBLIC OF |
|  |  |  |
| SangYun Kim | Seoul National University Bundang Hospital, Department of Neurology  82 gumi-ro, 173beo-gil, Bundang-gu  Seongnam-si, Gyeonggi-do 463-707  KOREA, REPUBLIC OF | Seoul National University Bundang Hospital  166 Gumi-ro, Bundang-gu  Seongnam-si, Gyeonggi-do 463-707  KOREA, REPUBLIC OF |
|  |  |  |
| Seong Hye Choi | Inha University Hospital, Department of Neurology  27, Inhang-ro, Jung-Gu  Incheon,  KOREA, REPUBLIC OF | Institutional Review Board of Inha University Hospital  A-F6, Jungsuk Building, 7-241, 3-Ga, Sinheong-Dong, Jung-Gu  Incheon, 400-711  KOREA, REPUBLIC OF |
|  |  |  |
| Seol-Heui Han | Konkuk University Medical Center, Department of Neurology  4-12 Hwayang-dong, Kwangjin-gu  Seoul, 143-914  KOREA, REPUBLIC OF | Institutional Review Board for Human Research, Konkuk University Medical Center  4-12 Hwayang-dong, Kwangjin-gu  Seoul, 143-914  KOREA, REPUBLIC OF |
|  |  |  |
| Kun-Woo Park | Korea University Anam Hospital  73, Inchon-ro, Seongbuk-gu,  Seoul, 136-705  KOREA, REPUBLIC OF | Korea University Anam Hospital IRB  73, Inchon-ro, Seongbuk-gu  Seoul, 136-705  KOREA, REPUBLIC OF |

## United States of America

| **Principal Investigator** | **Address(es)** | **Institutional Review Board or Ethics Committee Address(es)** |
| --- | --- | --- |
| Dr. Joel Steven Ross | Central Jersey Radiology  MRI Only  2128 Kings Highway & Route 35  Oakhurst, NJ 07755  UNITED STATES  Memory Enhancement Center of America, Inc.  2nd Floor  4 Industrial Way West  Eatontown, NJ 07724  UNITED STATES  Pharmacare USA  Drug Shipment Only  Suite B  95 Newfield Avenue  Edison, NJ 08837  UNITED STATES | Schulman Associates Institutional Review Board, Incorporated  4290 Glendale - Milford Road  Cincinnati, OH 45242  UNITED STATES |
|  |  |  |
| Dr. Beth Emmie Safirstein | MD Clinical  911 East Hallandale Beach Boulevard  Hallandale Beach, FL 33009  UNITED STATES | Schulman Associates Institutional Review Board, Incorporated  Suite 300  4445 Lake Forest Drive  Cincinnati, OH 45242  UNITED STATES |
|  |  |  |
| Dr. Franco Sicuro | DePaul Health Center  12303 DePaul Drive  St. Louis, MO 63044  UNITED STATES  Millennium Psychiatric Associates, LLC  Suite 230  777 Craig Road  Creve Coeur, MO 63141  UNITED STATES | Schulman Associates Institutional Review Board, Incorporated  Suite 300  4445 Lake Forest Drive  Cincinnati, OH 45242  UNITED STATES |
|  |  |  |
| Dr. Robert Enoch Litman | CBH Health, LLC  Suite 170  9605 Medical Center Drive  Rockville, MD 20850  UNITED STATES  Foers Medical Arts Pharmacy  8218 Wisconsin Avenue  Bethesda, MD 20814  UNITED STATES | Schulman Associates Institutional Review Board, Incorporated  4290 Glendale - Milford Road  Cincinnati, OH 45242  UNITED STATES |
|  |  |  |
| Dr. Robert Alan Riesenberg | Atlanta Center for Medical Research  811 Juniper Street Northeast  Atlanta, GA 30308  UNITED STATES | Schulman Associates Institutional Review Board, Incorporated  Suite 300  4445 Lake Forest Drive  Cincinnati, OH 45242  UNITED STATES |
|  |  |  |
| Dr. Louise Anne Taber | Pivotal Research Center  Suite 200  13128 North 94th Drive  Peoria, AZ 85381  UNITED STATES | Schulman Associates Institutional Review Board, Incorporated  4290 Glendale - Milford Road  Cincinnati, OH 45242  UNITED STATES |
|  |  |  |
| Dr. Howard Mark Waxman | Albert Einstein Medical Center  5501 Old York Road  Philadelphia, PA 19141  UNITED STATES  Belmont Center for Comprehensive Treatment  4200 Monument Road  Philadelphia, PA 19131-1689  UNITED STATES | Albert Einstein Healthcare Network IRB  5501 Old York Road  Philadelphia, PA 19141  UNITED STATES |
|  |  |  |
| Dr. John Paul Nardandrea Jr. | Advanced Imaging of Ocala  (MRI Only)  8150 SW SR 200  Ocala, FL 34481  UNITED STATES  Franck¿s Pharmacy  (Drug Shipment Only)  Suite B  202 Southwest 17th Street  Ocala, FL 34471  UNITED STATES  Munroe Regional Medical Center  (LP site only)  1500 SW 1st Avenue  Ocala, FL 34471  UNITED STATES  Renstar Medical Research  Suite B  104 Southeast First Avenue  Ocala, FL 34471  UNITED STATES  Renstar Medical Research  Suite 301  2405 Southeast 17th Street  Ocala, FL 34471  UNITED STATES  Renstar Medical Research  Suite 502  2405 Southeast 17th Street  Ocala, FL 34471  UNITED STATES | Schulman Associates Institutional Review Board, Incorporated  4445 Lake Forest Drive  Suite 300  Cincinnati, OH 45242  UNITED STATES |
|  |  |  |
| Dr. Phillip Michael Green | Borgess Medical Center  1521 Gull Road  Kalamazoo, MI 49048  UNITED STATES  Borgess Research Institute  NP 003  1521 Gull Road  Kalamazoo, MI 49048  UNITED STATES  Borgess Research Institute  Drug Shipment Address:  Suite 003  1717 Shaffer Street  Kalamazoo, MI 49048  UNITED STATES  KNI/Southwest Michigan Imaging Center, LLC  MRI Only:  1700 Gull Road  Kalamazoo, MI 49048  UNITED STATES | Schulman Associates Institutional Review Board, Incorporated  4290 Glendale - Milford Road  Cincinnati, OH 45242  UNITED STATES |
|  |  |  |
| Dr. Joseph Allen Kwentus | Brentwood Behavioral Healthcare  3531 Lakeland Drive  Flowood, MS 39232  UNITED STATES  Marty's Pharmacy  1042 River Oaks Drive  Flowood, MS 39232  UNITED STATES  Precise Research Centers  Brentwood Plaza - Suite 1060  3531 Lakeland Drive  Flowood, MS 39232  UNITED STATES | Schulman Associates Institutional Review Board, Incorporated  Suite 300  4445 Lake Forest Drive  Cincinnati, OH 45242  UNITED STATES |
|  |  |  |
| Dr. Mohammed Abdul Bari | Early Phase Investigational Center  704 East Grand Avenue  Escondido, CA 92025  UNITED STATES  Synergy Clinical Research Center of Escondido  710 East Grand Avenue  Escondido, CA 92025  UNITED STATES | Schulman Associates Institutional Review Board, Incorporated  4290 Glendale - Milford Road  Cincinnati, OH 45242  UNITED STATES |
|  |  |  |
